# Acai Extract Transiently Upregulates Erythropoietin by Inducing a Renal Hypoxic Condition in Mice

**DOI:** 10.3390/nu12020533

**Published:** 2020-02-19

**Authors:** Shuichi Shibuya, Toshihiko Toda, Yusuke Ozawa, Mario Jose Villegas Yata, Takahiko Shimizu

**Affiliations:** 1 Aging Stress Response Research Project Team, National Center for Geriatrics and Gerontology, 7-430 Morioka-cho, Obu, Aichi 474-8511, Japan; s-shibuya@ncgg.go.jp; 2 Department of Endocrinology, Hematology and Gerontology, Chiba University Graduate School of Medicine, 1-8-1 Inohana, Chuo-ku, Chiba 260-8670, Japan; hik_toda-jac@proteome.jp (T.T.); ozawayusuke3@gmail.com (Y.O.); 3 FRUTA FRUTA, Inc., 3-3 Kandajimbo-cho, Chiyoda-ku, Tokyo 101-0051, Japan

**Keywords:** acai, erythropoiesis, erythropoietin

## Abstract

Acai (*Euterpe oleracea* Mart. Palmae, Arecaceae) is a palm plant native to the Brazilian Amazon. It contains many nutrients, such as polyphenols, iron, vitamin E, and unsaturated fatty acids, so in recent years, many of the antioxidant and anti-inflammatory effects of acai have been reported. However, the effects of acai on hematopoiesis have not been investigated yet. In the present study, we administered acai extract to mice and evaluated its hematopoietic effects. Acai treatment significantly increased the erythrocytes, hemoglobin, and hematocrit contents compared to controls for four days. Then, we examined the hematopoietic-related markers following a single injection. Acai administration significantly increased the levels of the hematopoietic-related hormone erythropoietin in blood compared to controls and also transiently upregulated the gene expression of *Epo* in the kidney. Furthermore, in the mice treated with acai extract, the kidneys were positively stained with the hypoxic probe pimonidazole in comparison to the controls. These results demonstrated that acai increases the erythropoietin expression via hypoxic action in the kidney. Acai can be expected to improve motility through hematopoiesis.

## 1. Introduction

To maintain oxygen homeostasis, mammals have hematopoietic regulatory mechanisms, including erythropoiesis. Erythropoietin (EPO) is a hematological factor mainly expressed in the kidney in adults [1]. EPO is induced under conditions of reduced oxygen levels, as well as blood loss [2]. The *Epo* transcription is regulated by hypoxia-inducible transcription factors (HIFs), which have two oxygen-responsive sites associated with prolyl hydroxylase and lead to degradation by ubiquitination under normoxia [3]. This evidence demonstrates that the redox states on renal proteins containing HIF are potential indicators of erythropoiesis in adult mammals.

Acai (*Euterpe oleracea* Mart. Palmae, Arecaceae) is a large palm plant found in the northern region of South America, called the “Amazon”, in Brazil. Acai berries have a high polyphenol content, including anthocyanins, such as cyanidine-3-glucoside (C3Glc), cyanidine-3-diglucoside, and cyanidin-3-rutinoside, which contribute to antioxidant activity [4]. In several rodent studies, the benefits of acai intervention have been reported to include improving cardiac dysfunction following myocardial infarction [5], protection from diet-induced obesity [6] and hepatic steatosis [7], prevention of brain oxidative damage [8], and modulation of age-related hippocampal inflammation [9]. Acai intake is also expected to be a useful therapeutic strategy for chronic kidney disease with oxidative stress, inflammation, and dysbiosis [10]. However, no studies have determined the erythropoietic effect of acai on renal redox alteration.

In the present study, in order to clarify the erythropoietic action of acai, we administered acai extract to mice and examined the relationship between the erythropoietic factors and the redox change in the kidney.

## 2. Materials and Methods

### 2.1. Animals

C57BL/6NCrSlc mice were purchased from Japan SLC (Shizuoka, Japan) and inbred in our own cohorts. The animals were housed under a 12-h light/dark cycle and fed an MF diet (Oriental Yeast Co., Ltd., Tokyo, Japan) ad libitum. The mice were maintained and studied according to the protocols approved by the Animal Care Committee of the Chiba University. 

### 2.2. Administration

Acai extract (Table 1, Lot. 171115 and 180622) provided by FRUTA FRUTA, Inc. (Tokyo, Japan) was made by finely grinding whole fruit and filtrating with a #30 strainer. The extract was orally administered at 10 mL/kg/day via gavage to mice 1 time (*n* = 8) and for four days (*n* = 4) at 12–16 weeks of age. C3Glc (NS380102) was purchased from Nagara Science (Gifu, Japan). ASP1517 (roxadustat, #15294) was purchased from Cayman Chemical (Ann Arbor, MI, USA). The water-dissolved C3Glc (50 mg/kg, *n* = 6) and 0.5% carboxymethyl cellulose-suspended ASP1517 (80 mg/kg, *n* = 7) were administered orally once to littermate mice of the acai-treated cohort. The study was performed using the blood and kidney tissue of animals collected under anesthesia 2–3 h after the final administration.

### 2.3. Histology

To evaluate the tissue hypoxic area, Hypoxyprobe^TM^-1 Omni (Hypoxyprobe, Inc., Burlington, MA, USA) was used [11]. Mice were sacrificed 15 min after being intraperitoneally injected with anesthetic and 60 mg/kg of pimonidazole. The kidney was fixed in a 4% paraformaldehyde phosphate buffered saline (PBS) (Nacalai Tesque, Inc., Kyoto, Japan) and embedded in paraffin. The rehydrated sections were antigen retrieved with 10 mM citrate buffer (pH 6.0, with 0.05% Tween 20) at 95 °C for 30 min, washed with PBS containing 0.1% Tween 20 three times, and intrinsic peroxidases were quenched with 3% H_2_O_2_ for 30 min. We performed blocking with 3% goat serum or Blocking One Histo (Nacalai Tesque) and then treated samples with 1:200 or 1:20 diluted anti-pimonidazole antibody (Hypoxyprobe, Inc.). The pimonidazole-positive area was evaluated by the QWin V3 imaging software program (Leica, Wetzlar, Germany) or a BZ-X710 analyzer (Olympus Co., Tokyo, Japan) using the ABC staining kit (Vector Labs., Inc., Burlingame, CA, USA) or the FITC-fluorescence method (goat anti-rabbit antibody #AP307F, Sigma (St. Louis, MO, USA) and Fluoro-KEEPER Antifade Reagent with DAPI #12745-74; Nacalai Tesque), respectively.

### 2.4. Measurement of EPO

The plasma EPO level was measured using the Mouse Erythropoietin Quantikine ELISA kit (#MEP00B; R&D Systems, Inc., Minneapolis, MN, USA) according to the manufacturer’s instructions. In brief, thawed plasma was diluted 2-fold by calibrator diluents and then incubated on an antibody-coated microplate with each volume of assay diluents for 2 h using an orbital shaker. Washed wells were treated with antiserum conjugate for 2 h and with a substrate mixture for 30 min. The optical density measured at 450 and 540 nm was analyzed with the standards curve using the 4-parameter logistic curve-fit.

### 2.5. Quantitative Polymerase Chain Reaction (PCR)

Total RNA was extracted from the kidney using the RNAlater (Thermo Fisher Scientific, Waltham, MA, USA) and the Sepasol-RNA I Super G reagent (Nacalai Tesque) according to the manufacturer’s instructions. The cDNA was synthesized from 1 mg total RNA using ReverTra Ace qPCR RT Master Mix (Toyobo, Osaka, Japan). Real-time PCR was performed with the SsoAdvanced SYBR Green Supermix (Bio-Rad, Hercules, CA, USA) on a Mini-Opticon (Bio-Rad) according to the manufacturer’s protocols. All data were normalized to the level of the housekeeping gene β-Actin/*Actb*. The following primers were used for the analysis: *Actb*, forward, 5′-GCC CTA GGC ACC AGG GTG TGA-3′, and reverse, 5′-TCC TCA GGG GCC ACA CGC A-3′; *Epo*, forward, 5′-TCA TCT GCG ACA GTC GAG TTC TG-3′, and reverse, 5′-GGT ATC TGG AGG CGA CAT CAA TTC-3′; and *Vegfa*, forward, 5′-GCA GCT TGA GTT AAA CGA ACG-3′, and reverse, 5′-GGT TCC CGA AAC CCT GAG-3′.

### 2.6. Hematological Cytometry

The number of red blood cells and leukocytes, hemoglobin levels, and hematocrit levels were measured by the Oriental Yeast hematology analyzing service (Tokyo, Japan).

### 2.7. Statistical Analyses

The statistical analyses were performed using the Student’s *t*-test for comparisons between two groups and a one-way analysis of variance/Tukey’s test for comparisons of three or more groups. Differences between the data were considered significant when the p-values were less than 0.05. All data are expressed as the mean ± standard deviation (SD).

## 3. Results

### 3.1. Acai Extract Alters Hematological Parameters

First, to investigate the hematological effects, we administered acai extract and measured the hematological parameters in mice. Treatment with acai extract significantly increased the erythrocyte content (RBC), hemoglobin level (HGB), and hematocrit level (HCT) but not the leukocyte content (WBC) or reticulocyte count (RET) in blood for four days (Table 2). Acai extract also induced no significant change in the erythrocyte mean cell volume (MCV), mean cell hemoglobin (MCH), mean cell hemoglobin concentration (MCHC), or platelet content (PLT) (Table 2). These results suggest that acai induced a hemopoietic effect or suppressed red blood cell degradation.

### 3.2. Acai Extract Acutely Upregulates the EPO Contents in Blood

**Figure 1:**
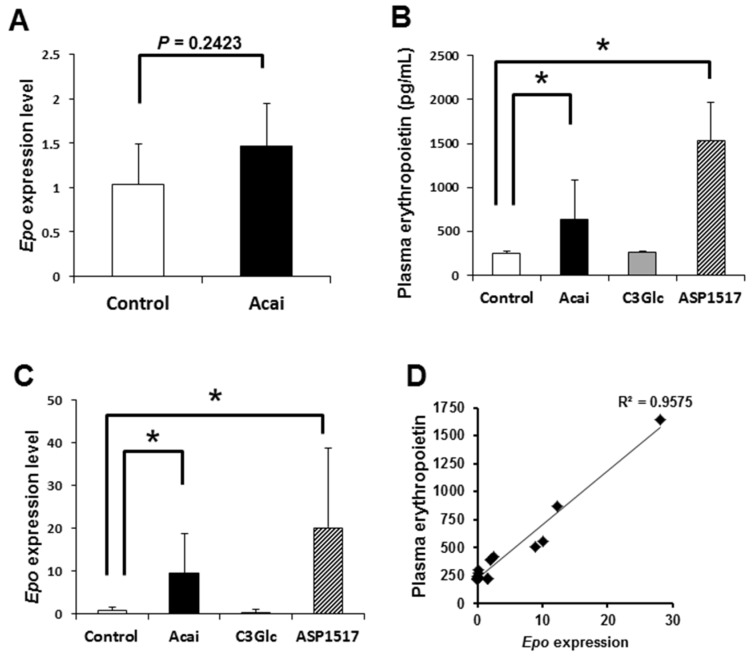
Acai extract upregulates both the plasma erythropoietin (EPO) concentration and kidney *Epo* expression. (**A**). The relative *Epo* transcript level in the kidney after the oral administration of acai extract (10 g/kg) dairy for four days. * *P* < 0.05 by *t*-test. (**B**) The plasma EPO concentration in mice 2–3 h after the oral administration of acai extract (10 g/kg), C3Glc (50 mg/kg), and ASP1517 (80 mg/kg). (**C**) Relative *Epo* transcript levels in kidney 2–3 h after the oral administration of acai extract (10 g/kg), C3Glc (50 mg/kg), and ASP1517 (80 mg/kg). (**D**) Relationship between the plasma EPO concentration and *Epo* transcription in kidney. Error bars indicate the standard deviation. * *P* < 0.05 by an ANOVA/Tukey’s test.

EPO is the principle regulator of red blood cell production [12]. In general, the *Epo* expression is transiently stimulated by a hypoxic condition [2]. After four days of administration of acai, the renal *Epo* expression showed a slight increase (Figure 1A). In this context, we performed a transient experiment, administering acai extract to mice and measuring the EPO contents in plasma 2–3 h after treatment. The acai treatment caused a significant increase in the plasma EPO level compared with vehicle control (Figure 1B). Furthermore, acai upregulated the *Epo* transcript level in the kidney compared with the control (Figure 1C). The erythropoiesis inducer roxadustat (ASP1517), which is also used to treat renal anemia, also upregulated both the EPO contents in plasma and the *Epo* transcript level in kidney (Figure 1B,C). In contrast to acai, the administration of C3Glc caused no significant change in either the EPO contents or the *Epo* level (Figure 1B,C). Furthermore, the relationship between the plasma EPO concentration and the kidney *Epo* transcript level was positive (Figure 1D). These results suggest that acai extract transcriptionally induced EPO production in the kidney.

### 3.3. Acai Extract Induces a Renal Hypoxic Condition

**Figure 2:**
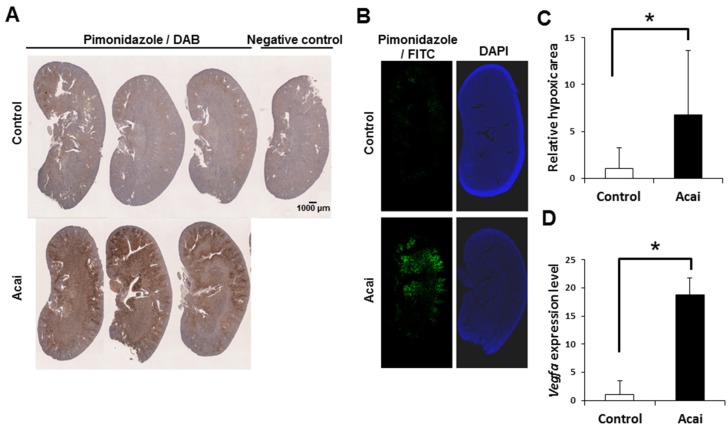
Acai extract induces a hypoxic reaction in kidney. (**A**–**C**) The pimonidazole-positive hypoxic area in kidney sections 2–3 h after the oral administration of acai extract (10 g/kg) was detected by 3,3′-diaminobenzidine (**A**) and fluorescein isothiocyanate (**B**,**C**). (**D**) Relative *Vegfa* transcript level in the kidney 2–3 h after acai extract. Scale bar denotes 1 mm. Error bar indicates the standard deviation. * *P* < 0.05 by *t*-test.

Finally, to investigate the relationship between EPO induction and renal hypoxia under acai administration, we administered the hypoxic probe pimonidazole to mice and histologically detected renal hypoxia. By immunostaining with pimonidazole, which accumulates in hypoxic areas, acai extract was shown to induce renal hypoxia (Figure 2A). A quantitative analysis with the fluorescence intensity of pimonidazole staining showed that acai created a significantly larger hypoxic area than was seen in the controls (Figure 2B,C). Furthermore, acai induced renal hypoxia mainly at the corticomedullary junction where erythropoietin is produced (Figure 2A,B). Along with hypoxia induction, acai also increased the expression of *Vegfa*, which located downstream of HIF, in the kidney (Figure 2D). These results suggest that renal erythropoietin production caused by acai depends on the hypoxic reaction in the kidney.

## 4. Discussion

Since EPO, a hematopoietic hormone that controls erythropoiesis, is produced in response to tissue hypoxia, athletes often incorporate high-altitude training. The induction of EPO expression mainly involves the transcription factor HIF induced by hypoxia [3]. Under hypoxic conditions, degradation of HIF is inhibited by ubiquitination due to the suppression of proline hydroxylase, thereby promoting hematopoiesis [3]. ASP1517 induces the production of erythrocytes via the prevention of HIF degradation by inhibiting the proline hydroxylase [13]. Actually, the remarkable induction of renal hypoxia by acai suggests the HIF-mediated *Epo* and *Vegfa* upregulation (Figure 1 and Figure 2).

In our study, acai administration did not increase RET, which is in contrast to the increases it induced in erythropoietin and HCT (Table 2). Testosterone supplementation improved anemia in aged mice without increasing the number of reticulocytes, suggesting the contributions of an increase in erythrocyte-related genes in spleen and the normalization of iron homeostasis [14]. Since acai contains a large amount of iron, it can also increase the ferritin and iron contents, leading to iron-related hematopoiesis. As EPO is transiently induced by hypoxic conditions [2], the upregulation of EPO by acai may affect acute erythropoiesis. These findings suggest that, in contrast to the HIF-mediated erythropoiesis mechanism of ASP1517, acai may exert multiple hematopoietic mechanisms. Since the combination of EPO-stimulating and iron agents is an important anemia management strategy for patients undergoing hemodialysis [15], acai has potential applications in therapy for various types of anemia. We need to analyze the mechanisms underlying the hematopoietic effect of acai more strictly in future studies.

Acai contains large amounts of iron, various polyphenols, and long-chain fatty acids, such as oleate, palmitate and linoleate (Table 1), suggesting various beneficial effects for anemia. The monounsaturated oleic acid depresses the cytokine expression in vitro [16]. Stoner et al. reported that acai reduced the serum IL-5 and IL-8 levels in rats [17]. Anemia in the elderly population is caused by the increased expression of proinflammatory cytokines [18]. Acai may protect against anemia by reducing the levels of inflammatory cytokines increased by aging and various stressors. Furthermore, acai treatment attenuated renal ischemia/reperfusion injury via protection against renal oxidative stress in diabetic and spontaneously hypertensive rats [19,20]. In a rat study, acai treatment significantly decreased the 8-isoprostane immunostaining level and thiobarbituric acid reactive substances level in the kidney [20], suggesting the transition from an oxidative to a reductive condition in the kidney. These findings show that acai induces erythropoietic action via an altered redox status in the kidney. Mitochondrial angiotensin II receptors regulate oxygen consumption in kidney mitochondria [21], suggesting a deep association with the renal status and oxygen consumption. Hernandez-Vargas et al. reported that the suppressors of cytokine signaling (SOCS) family negatively regulated the angiotensin II-activated signal pathway, including JNK, resulting in a decreased renal oxygen consumption [22]. AICAR, an AMPK activator, decreased the renal oxygen consumption via the modulation of SOCS1 [23]. In this context, acai may inhibit the SOCS family, leading to the activation of the angiotensin II-mediated pathway associated with increased oxygen consumption and hypoxia in the kidney.

Polyphenols protect against reactive oxygen species-induced hemolysis via increased red blood cell integrity associated with the inhibition of lipid peroxidation [24,25,26,27]. Similarly, red cabbage extracts rich in anthocyanins rescue oxidative hemolysis in streptozotocin-induced diabetes [28]. Anthocyanins also exert antisickling activity by stabilizing red blood cells and their membranes and inhibiting polymerization on hemoglobin S [29,30]. However, C3Glc alone did not alter the erythropoietin or *Epo* levels (Figure 2), suggesting that not only polyphenols but also iron and fatty acids of acai contribute to erythropoiesis.

Acai showed no toxicity in experimental models [31,32,33,34,35,36] nor any significant differences in the body weight or food consumption [17,31,32,36], suggesting potential applications in the prevention of various disease. Conversely, Mn, which is abundant in acai, suppresses Fe absorption, suggesting a risk for anemia [37]. The kidneys are prone to hypoxia because of their high energy consumption [38]. Chronic renal hypoxia is a final common pathway to end-stage renal failure, resulting in an irreversible decline in the renal function [39,40]. In a study of hypertensive rats, acai treatment for 45 days decreased the creatinine contents in the serum and urine, suggesting a protective effect on the kidney [20]. Since we only performed the acai trials for up to four days, a more detailed evaluation of the effects of longer-term treatment on the hematopoietic activity and renal function is needed. EPO formulations are presently banned as a doping substance. The erythropoietic mechanism of acai via EPO must be further clarified, and its use in sports needs to be carefully discussed.

In summary, acai induces an erythropoietic effect associated with renal hypoxia. These findings provide valuable insight into the potential utility of acai for future research on hematopoiesis in humans.

## Figures and Tables

**Table 1:** Contents in 100 g of acai extract.

Contents		Fatty Acids	(%)
Energy intake	82.0 kcal	Palmitic acid (C16:0)	22.5
Protein	1.4 g	Palmitoleic acid (C16:1)	3.3
Total lipids	6.9 g	Stearic acid (C18:0)	1.9
Carbohydrates	2.0 g	Oleic acid (C18:1)	61.2
Dietary fiber	3.3 g	Linoleic acid (C18:2)	10.6
Total polyphenols	390 mg	Linolenic acid (C18:3)	0.5
Iron	1.0 mg		

**Table 2:** Effect of acai extract on the hematological parameters in mice (*n* = 4).

	Control	10 g/kg of Acai for Four Days
WBC (/µL)	2500 ± 804	2275 ± 660
RBC (×10^4^/µL)	876 ± 11	931 ± 12 *
HGB (g/dL)	14.1 ± 0.5	14.9 ± 0.4 *
HCT (%)	47.6 ± 0.6	51.1 ± 1.4 *
MCV (fL)	54.4 ± 0.7	54.9 ± 0.8
MCH (pg)	16.0 ± 0.5	16.0 ± 0.4
MCHC (%)	29.5 ± 0.8	29.3 ± 0.9
PLT (×10^4^/µL)	95 ± 5	97 ± 13
RET (‰)	27.8 ± 4.8	23.0 ± 1.2

Hematological parameters in blood treated with acai extract (10 g/kg) daily for four days orally. WBC: Leukocyte content; RBC: Erythrocyte content; HGB: Hemoglobin; HCT: Hematocrit; MCV: Mean cell volume; MCH: Mean cell hemoglobin; MCHC: Mean cell hemoglobin concentration; PLT: Platelet content; RET: Reticulocytes. * *P* < 0.05 by *t*-test.
